# Supplementary material for: Snapshots of a shrinking partner: Genome reduction in Serratia symbiotica
Source: Sci Rep. 2016 Sep 7;6:32590. doi: 10.1038/srep32590 (PMC5013485; doi:10.1038/srep32590)
Supplement: Supplementary Information [file srep32590-s1.pdf]

Snapshots of a shrinking partner: Genome reduction in *Serratia symbiotica*

Alejandro Manzano-Marín[1,*] **and Amparo Latorre**[1,2,*]

[1]Institut Cavanilles de Biodiversitat I Biologia Evolutiva - Universitat de València, Genética Evolutiva, Paterna, 46980, Spain
[2]Fundación para el Fomento de la Investigación Sanitaria y Biomédica de la Communitat Valenciana (FISABIO), Genómica y Salud, València, 46020, Spain
[*]alejandro.manzano@uv.es
[*]amparo.latorre@uv.es

Supplementary information

Supplementary figures

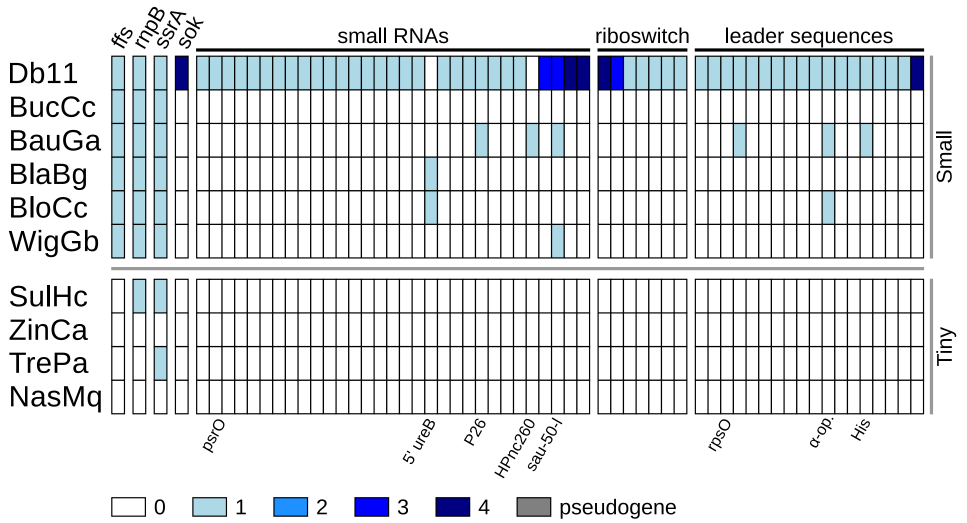

Figure S1. Decay of RNA features in highly-reduced genomes. Colour-coded diagram showing the decay of RNA features in different highly-reduced genomes comparing against free-living Db11. On top of the matrix, gene names (for the first four columns) and RNA categories (for the rest) are indicated. On the bottom of the matrix, feature names are indicated for those features retained in highly-reduced genomes. Small and tiny genomes are separated by a grey bar. BucCc= *Buchnera* from *Cinara cedri*, BauGa=*Baumannia* from *Graphocephala atropunctata*, BlaBg=*Blattabacterium* from *Blattella germanica*, BloCc=*Blochmannia* from *Camponotus chromaiodes*, WigGb=*Wigglesworthia* from *Glossina brevipalpis*, SulHc=*Sulcia* from *Homalodisca vitripennis*, ZinCa=*Zinderia* from *Clastoptera arizonana*, TrePa=*Tremblaya phenacola* from *Phenacoccus avenae*, NasMq=*Nasuia* from *Macrosteles quadripunctulatus*.

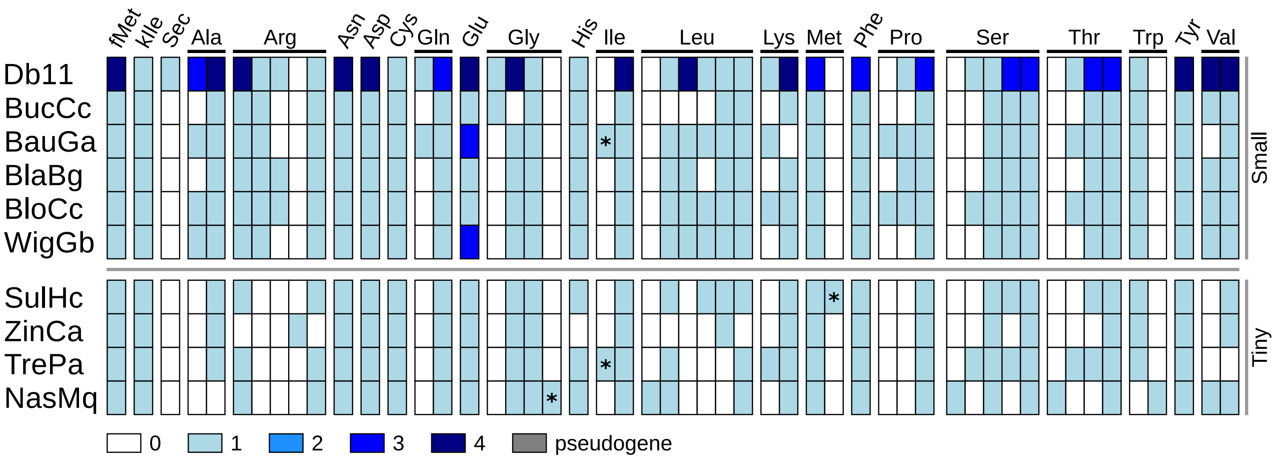

Figure S2. **Decay of tRNA features in highly-reduced genomes.** Colour-coded diagram showing the decay of tRNA features in different highly-reduced genomes. On the top of the matrix, aminoacyl charging potential for each tRNA species (as inferred by TFAM). Each column represents a different anticodon. fMet=*N*-Formylmethionine, kIle= lysylated isoleucine. Asterisks indicate putative codon reassignments. Small and tiny genomes are separated by a grey bar. BucCc= *Buchnera* from *Cinara cedri*, BauGa=*Baumannia* from *Graphocephala atropunctata*, BlaBg=*Blattabacterium* from *Blattella germanica*, BloCc=*Blochmannia* from *Camponotus chromaiodes*, WigGb=*Wigglesworthia* from *Glossina brevipalpis*, SulHc=*Sulcia* from *Homalodisca vitripennis*, ZinCa=*Zinderia* from *Clastoptera arizonana*, TrePa=*Tremblaya phenacola* from *Phenacoccus avenae*, NasMq=*Nasuia* from *Macrosteles quadripunctulatus*.

Supplementary tables

	Mobile element type		
**Organism**	**IS**	**Group II intron**	**Tn*TIR***
Db11	9	0	1
SAf	306	27	2
SAp	131*	2*	13
SCt	405	12	9
SCc	0	0	0
STs	0	0	0

Table S1. **Mobile elements by type in *S. symbiotica* and *S. marcescens* Db11.** *Absolute counts may be underestimated given the highly-fragmented genome assembly.

Acknowledgements

This work has been funded by the Ministerio de Economía y Competitividad (Spain) co-financed by FEDER funds [BFU2015-64322-C2-1-R to A.L.]; the European Commission [Marie Curie FP7 PITN-GA-2010-264774-SYMBIOMICS to A.M.M]; and the Consejo Nacional de Ciencia y Tecnología (Mexico) [Doctoral scholarship CONACYT 327211/381508 to A.M.M.]. The funders had no role in study design, data collection and analysis, decision to publish, or preparation of the manuscript.

Author contributions statement

A.M.M. and A.L. conceived and designed the study. A.M.M. analysed the data. A.M.M. and A.L. wrote, reviewed, and approved the final version of the manuscript.

Competing financial interests

The authors declare no competing financial interests.
